# Does an educative approach work? A reflective case study of how two Australian higher education Enabling programs support students and staff uphold a responsible culture of academic integrity

**DOI:** 10.1007/s40979-021-00099-1

**Published:** 2022-03-01

**Authors:** Anthea Fudge, Tamra Ulpen, Snjezana Bilic, Michelle Picard, Carol Carter

**Affiliations:** 1 University of South Australia, UniSA College: Education Futures, Adelaide, SA 5000 Australia; 2 Murdoch University, Perth, WA 6150 Australia; 3 Curtin University, Perth, WA 6102 Australia

**Keywords:** Academic integrity, Enabling programs, Academic culture, Student support, Institutional change, Higher education, Australia

## Abstract

**Introduction:**

Enabling education programs, otherwise known as Foundation Studies or Preparatory programs, provide pathways for students typically under-represented in higher education. Students in Enabling programs often face distinct challenges in their induction to academic culture which can implicate them in cases of misconduct. This case study addresses a gap in the enabling literature reporting on how a culture of academic integrity can be developed for students and staff in these programs through an educative approach.

**Case description:**

This paper outlines how an educative approach to academic integrity is implemented within the Enabling programs of two Australian universities.

**Discussion and reflection:**

This case study reflects upon an approach which makes specific reference to the key elements of ‘support’, ‘approach’ and ‘responsibility’ as highlighted in Bretag and Mahmud’s seminal paper. The paper reports a reduction in misconduct cases at the two institutions suggesting a positive correlation between the interventions and students’ understanding of ethical academic practice. This study reflects upon practitioner experiences with academic integrity investigations to evaluate the effectiveness of this approach.

**Conclusions:**

The authors show that it is possible to ensure academic integrity practices and values are upheld within a supportive learning environment appropriate to a students’ level of study.

## Introduction

Across the globe, higher education (HE) institutions strive to demonstrate educational quality in their teaching, research, and interactions with the community. Academic misconduct undermines the intellectual reputation of universities and academics and diminishes the respect of scientists and researchers in local communities. Preventing, detecting, and responding to academic integrity (AI) breaches has become increasingly complex due to a range of Internet tools and services and the scourge of assignment sharing and contract cheating sites. Governments and universities have gone to great lengths to prevent AI breaches. For example, in Australia, the Tertiary Education Quality and Standards Agency (TEQSA) Amendment (Prohibiting Academic Cheating Services) Bill read in 2019 by the Minister of Education provides a raft of recommendations and sanctions against third-party academic cheating services in HE. These include criminal and civil penalties to be enforced. However, despite wanting to send a strong warning about engaging with these cheating services, in the Minister’s own words, it is important “to take an educative approach in the first instance”. Detection of all AI breaches is virtually impossible. Therefore, educating rather than just punishing students who are in the process of learning how to operate in a HE context is a more successful strategy in preventing academic misconduct. The literature has emphasised the importance of creating a culture of AI that includes ethical values at all levels of higher education (Bretag and Mahmud 2016; Gow 2014; Morris and Carroll 2015). Morris and Carroll (2015) contend that the greatest AI impact on student behaviour can be achieved if ethical values are learned through practical experiences holistically addressed in a supportive learning environment. There are multiple policies and practices such as text-matching software for detection of plagiarism, educational interventions and policies within institutions and increasingly at a government level that strive to prevent AI breaches. However, these tend to have little impact without a culture of integrity and an educative approach appropriate to the students’ level of study.

Students studying within ‘enabling’ or ‘foundation studies’ programs require support in the process of enculturation into HE and in their learning of ethical academic practices. The official definition of an Enabling program according to the Higher Education Support Act (2003), is “a course of instruction provided to a person for the purpose of enabling the person to undertake a course leading to a higher education award” (Department of the Attorney General 2003, p. 215). Yet, a more common understanding is a course that endeavours to teach and provide students with foundational academic skills in preparation for their transition to undergraduate study (Pitman et al. 2016). Enabling programs are embedded within the HE institutions in Australia, but the enabling space is different to traditional HE because of the diversity of student cohort (Bennett et al. 2016). Students who enrol in Enabling programs are either early school leavers or have had limited success in their final years of secondary education. The enabling sector grew exponentially across Australia in the past decade with enabling educators terming this distinctive inclusive approach to teaching students in preparatory programmes as enabling pedagogy (see Hockings 2010; Kift et al. 2010; Bennett et al. 2016; Motta and Bennett 2018). Enabling pedagogy draws upon the history of progressive pedagogies and maintains a focus on social justice and empowering students (Stokes 2014; Bennett et al. 2016). In comparison to credit-bearing undergraduate programs, Enabling programs are distinct in that there are generally no, or minimal entry requirements imposed. They are funded by the Commonwealth Government so that students do not incur tuition fees. These distinct characteristics of Enabling programs facilitate access to equity groups who may not have the financial capacity or previous educational experience for entry via traditional pathways. Removing entry requirements allows a diversity of students to enrol in the programs. The students represent a vast range of educational, cultural, linguistic and socio-economic backgrounds, and therefore their learning needs can be quite specific and require various support as highlighted in the brief literature review below.

## Literature review

### The enabling context – the widening participation agenda

Enabling programs play an integral role in widening participation in HE for a range of social demographic groups typically under-represented in the sector. Providing opportunity to experience a tertiary education within a socially inclusive system was a vision of the landmark Bradley Review (Bradley et al. 2008). This review was instrumental in HE reform “to increase the number of 25-34 year-olds holding a bachelor degree to 40% by 2025; and to increase the participation rate of people from low Socio-economic status (SES) backgrounds to 20% by 2020” (Australian Government 2009, pp. 12–13). Not only did these targets call for a more egalitarian system but also could have potential impacts in developing the nation as a knowledge economy.

### The diversity of the student cohort - challenges and distinctive needs

The diverse cohort of students in Enabling programs necessitate pedagogical and social support that meets their needs. Research on enabling pedagogies and curriculum has highlighted certain features of the enabling pathway environment that foster a sense of belonging and capability despite previous negative experiences (Burke et al. 2016). These include scaffolding approaches, explicit explanation, peer-mentoring, counselling and additional academic support embedded in the programs (Picard et al. 2018; Hellmundt and Baker 2017; Lane and Sharp 2014; Hodges et al. 2013; Hrasky and Kronenberg 2011).

Acculturation to a new academic environment can be challenging, but even more so for students who have past negative and/or limited educational experience. This enabling cohort must navigate several challenges such as language proficiency barriers and an unfamiliarity with academic literacies, discourses and practices which can compromise their ability to follow AI protocol. We acknowledge three specific challenges (a lack of university cultural capital (Bordieu 1977), inefficient time management skills, and language and literacy barriers) which can have a direct effect on an enabling student’s ability to understand and abide by the practices and principles of AI. Because of these challenges, enabling cohorts in particular benefit from an educative approach. Acquiring the cultural capital to navigate university systems can be quite a daunting experience for commencing students. This is particularly true for those who are the first in their family to attend university and/or come from areas of socio-economic disadvantage. Ball and Vincent (1998, pp. 380–381) propose the concepts of “hot” or “cold” knowledge to explain the domains of a student’s cultural capital. “Hot” knowledge (cultural capital) includes the perspectives, knowledge, cultural messaging and beliefs learnt and ingrained through family, upbringing and culture. “Cold” knowledge are unfamiliar messages communicated by institutions such as universities. Similarly, James, Krause and Jenkins (2010) found that first year students from low socio-economic backgrounds were more likely to be challenged by the cultural capital of the institution due to a lack of experience and therefore had difficulty understanding course content, university systems and teaching styles. This further reinforces Bourdieu’s (1977) theory of social reproduction showing schools to be institutions that perpetuate social inequalities. The reproduction of these inequalities is argued by Bourdieu to be facilitated by teachers’ pedagogic actions that promote the cultural capital of the dominant class by rewarding students who possess such capital and by penalising others who do not. Hattam, Stokes and Ulpen (2018) explain how:

this presents specific challenges for [E]nabling program students and other first-in-family students. How do new students with limited university contacts know about the structures in place and other norms of higher education? How do they know where to turn for assistance or unpack the complexities of university language and culture? (p. 6)

The “cold” knowledge can generate feelings of ‘imposter syndrome’ and repress a student’s attempt to acculturate into a new university environment. At this point, the responsibility of the institution comes into play to support and guide these students with AI principles, rather than assume they are familiar with the university’s expectations, discourse and academic practices. Naylor and Mifsud (2019) conducted a report exploring how institutions can address the systemic barriers which compound structural and cultural inequities experienced by equity group students and ultimately influence attrition rates. Using structural inequality as the theoretical framework, their investigation focused on how institutions may impede or facilitate students’ sense of belonging and enculturation into the new academic environment. Structural barriers might include using exclusive discourse in the learning space, privileging preferred communication styles and being inflexible with assessment and enrolment policies. Their research found that “removing or mitigating structural barriers appears likely to reap benefits in terms of retention, success and a positive student experience- particularly for students from equity groups” (p. 3). Hence, institutions need to recognise that students’ unfamiliarity with institutional structures and systems can compromise their compliance with AI norms and expectations. Breaches of AI might occur for example on account of inexperience with computer-generated similarity reports, an unfamiliarity with the institution’s preferred referencing system- let alone the specific referencing styles associated with the discipline they are enrolled in; a misunderstanding of collusion when the intention is simply to assist a friend; and cultural differences regarding the seriousness of exam protocol and procedures experienced in Australia compared to other countries. McKay and Devlin (2014, pp. 952–53) assert that students need to be “equipped with the tools and resources to help them gain the cultural and social capital required to play the game”.

In their transition to academic studies, enabling students can also encounter difficulties balancing a new study timetable with other competing demands such as family care responsibilities and work. The struggle to balance study and other life commitments in conjunction with understanding a new academic discourse and content can impact a students’ participation and engagement with course materials (Quinn and Wedding 2012). This dilemma can fuel feelings of anxiety and paralyse a student’s ability to prepare for assignments and meet deadlines. Consequently, the pressure of dealing with an excessive workload while trying to submit assignments on time can sway a student’s decision to plagiarise and/or collude. This ultimately leads to a breach of AI.

Another challenge which can have a direct effect on an enabling student’s ability to comply with AI practices is poor language and literacy proficiency. Open entry Enabling programs do not mandate language requirements, and thus students with a diversity of language proficiencies enrol in the programs (Cocks and Stokes 2012; 2013). Some students have only learnt English for 6 months since their arrival in Australia and are grappling with sentence structure, while others may have been privy to an earlier education but have little experience with academic literacies. This poses several issues with the teaching and learning of AI. Teaching staff acknowledge the need to not only teach AI literacies such as summarising, paraphrasing, researching, referencing, and synthesising, but also incorporate tuition in areas such as reading comprehension, sentence and paragraph writing within the curriculum (Murray 2011; Klinger and Murray 2012). There is a significant difference between learning sentence structure and being able to demonstrate command of more sophisticated literacies such as researching academic texts and synthesis. Therefore, many students with a limited command of the language, particularly those who speak English as an additional language or dialect (EAL/D), can fall into the trap of plagiarising and colluding (Bretag 2007). Therefore, it is reasonable to assume enabling students, particularly those with weaker language and literacy proficiencies, will experience complications and may inadvertently breach AI protocol.

### Enabling and educative approaches

Previously, Picard et al. (2018) highlighted the similarities between an educative approach to AI development as detailed by Bretag and Mahmud (2016) and the ‘enabling approach’ developed by educators supporting this cohort in the early stages of HE transition. Clear explicit instruction, scaffolding and modelling is required alongside formative opportunities to learn and make mistakes. It was also suggested that supports for AI and support-seeking behaviours should be normalised with staff taking an attitude of respectful guidance and flexibility to accommodate the diversity of the student cohort in their induction to academic culture (Picard et al. 2018). Although there are clear links between the ‘enabling approach’ and the ‘educative’ approach to AI, to date, no study has explored the impact of these approaches in practice. Hence this study aims to answer the following research question:

How can Enabling programs provide effective support to enhance students’ learning of AI and decrease the instances of academic misconduct through an educative approach?

## Case description: University of South Australia College and University of Newcastle, English Language and Foundation Studies Centre

In order to explore the research question above, this paper reports on a qualitative case study of two Australian university Enabling programs referred to as University of South Australia College (UCO) and, at the time of data collection, was called University of Newcastle, English Language and Foundation Studies Centre (UoNC) - now called the Pathways and Academic Learning Support Centre. The paper reviews academic misconduct case numbers and includes reflections of teaching staff involved in AI investigations over the periods of 2016 to 2021 (UCO) and 2018 to 2021 (UoNC). We, the authors, selected to describe these two Enabling program case studies since they reflect the range of Enabling programs available in Australian HE. Case study is a common methodology used in qualitative research (Rashid et al. 2019) that explores real-world phenomena within their natural context and uses a combination of qualitative and quantitative collection methods to increase the reliability of the research (Yin 2009). UoNC is one of the oldest and largest open-access Enabling foundation programs and UCO is another key Enabling program that provides these pathways providing both for credit and open-access non-credit programs. Prior to to this study, students in these programs encountered many of the AI issues highlighted in the literature review above. Since 2016 for UCO and 2018 for UoNC, both programs have systematically implemented an ‘educative’ approach in line with enabling principles.

We have all served as either Academic Integrity Officers (AIOs) or Student Academic Conduct Officers (SACOs) at one of the two HE institutions in this case study and therefore provide an insider’s view of the two case studies. At UCO, three AIOs manage and investigate referred AI cases. At UoNC, there are SACOs appointed for each faculty and school as opposed to the centralisation of AI matters. Two SACOs are responsible for AI issues in the Enabling programs that are the focus for the case study. However, the SACO processes and practices are also informed by the collective committee of SACOs and SACO chair from all faculites and schools.

As part of an ‘educative’ approach, we have implemented a suite of learning resources and investigation strategies designed to support and advance our students’ understanding of AI. This paper draws upon a combination of qualitative and quantitative data to reflect upon the effectiveness of the strategies we have implemented. The qualitative data includes reflections of our experiences in investigating misconduct cases and the impact of modifying our approach over time. Further, anecdotal student and staff feedback have provided valuable insight regarding our AI learning curricula resources and revised processes for management and investigation of cases. Triangulating this data with quantitative records of AI misconduct cases has allowed us to examine if our new approach has affected a reduction in AI cases. Further details of the programs and methods are provided below.

### Programs

UCO offers two programs of study: a one-year, Commonwealth supported Foundation Studies program and a two-year Diploma program streamed into seven disciplinary areas. The programs are primarily designed to prepare students with the requisite academic literacies needed for transition into undergraduate study. Students have the option to study full/part-time on the metropolitan campuses, at regional centers or online. Yearly enrolment numbers for 2020 were 953 Equivalent Full-Time Student Load (EFTSL) and in 2021 were 937 EFTSL.

UoNC also offers Commonwealth supported Foundation Studies programs which include a broad range of subjects across the undergraduate curriculum ranging from physics and engineering to Foundations of Nursing and Education. Students at this regional university can take these programs in a one-year full time mode, one to two-year part-time mode (face-to-face or online) or in a half-year intensive mode. There were 4263 enabling students enrolled in 2020 and 4201 in 2021. Students in the Enabling program at The University of Newcastle had the choice of approximately 35 (semester 1) and 45 (semester 2) Foundation subjects in 2020 of which they were expected to select 2 subjects per teaching period.

## Methods

While we have chosen to write a comprehensive paper based on both case studies, rather than a comparative one, the strategies we have adopted have been informed by close, collegial collaboration between the two universities. The case studies review the suite of resources and educative approaches adopted by each institution and their subsequent effect on the number of AI cases referred to the AIO and SACO for investigation.

### Data collection: University of South Australia College

In accordance with The University of South Australia's institutional policy, the AIOs, sought approval to use University AI data from 2016 to 2020 for external publication including this publication. The University’s Provost and Chief Academic Officer endorsed the request to obtain data from the University’s AI Database revealing the number of cases investigated at the College. Data were analysed to determine if our approach had affected the number of students referred for AI breaches at the College. Data were further examined to determine if these students were then investigated for repeat breaches in their undergraduate degree programs. A formal ethics application was not required for this data collection as all data has been deidentified. At UCO, we implemented our new approach in 2016. Prior to this, challenges were encountered with managing the number of cases referred for investigation. From 2011 to 2014 only one of the three authors from UCO acted as AIO to service the student cohort. With a steady, yearly increase in program enrolments, the number of AI breaches grew at a significant rate. Consequently, the AIO met with approximately 20–30 students per week during peak assessment periods to investigate possible breaches in AI policy. In 2015, the two other UCO’s authors joined the AIO team, but the number of cases remained high. It was evident that students were struggling with a range of literacies including paraphrasing, referencing and utilizing the *Turnitin* report. In most instances the investigation found students unintentionally breached the AI policy due to unfamiliarity with academic conventions and low language proficiency. As previous research has shown (Park 2003; Devlin and Gray 2007; Perry 2010), acquiring the cultural capital of the academy is challenging for students in Enabling programs and first year undergraduate students. Navigating these challenges provides a reasonable cause for why some students breach policy. Helping students in their transition to university study necessitates responsive supports such as embedding AI learning resources and modules into curricula.

Another problem we encountered at UCO was students’ reluctance to take part in the AI investigation process. A growing number of students were not responding to AI meeting invitations indicating they were disengaged and feared punitive outcomes. It was clear we needed to examine our practice to address these concerns and assure students that engaging with AI processes is a part of their overall university experience. Hence, we developed a video resource and adjusted policy letters with more tentative language to reassure students we take an educative approach in the investigation meetings.

### Data collection: University of Newcastle, English Language and Foundation Studies Centre 

A systematic ‘educative’ approach was implemented from the beginning of 2018 at UoNC. Prior to this date, AI cases among enabling students had been managed independently within the Centre and anecdotal evidence suggested that enabling educators were reluctant to report misconduct cases among their students so as not to discourage them from participating in HE. However, once they started their first year studies, there was a perception among some academic staff that AI breaches were more prevalent among the Centre’s student alumni than the University student cohort as a whole. Therefore, it was important to ensure that the approach taken within UoNC was consistent with the The University of Newcastle approach in general, yet at the same time provided a supportive ‘enabling’ approach. We obtained data for UoNC (collected from January 2018 to June 2021) from the SACOs records, Teaching and Learning Committee reports, and oral and written feedback from staff at various workshops. Permission was obtained from the Learning and Teaching Committtee to use the data generated from the SACO reports at The University of Newcastle in this article. Formal ethics clearance was not required as all data was deidentified. The authors, who had also worked as SACOs at UoNC have attempted to maintain objectivity and avoid bias in our data collection. This was achieved because the student data was obtained from a case reporting database and included cases from SACO officers not part of the writing team. Secondly, one of the authors, originally from The University of Newcastle, was not part of the workshop processes and collecting oral and written feedback and consequently could take a more objective view in analysing the data.

Three factors prompted The University of Newcastle SACOs decisions to make changes to their approach to AI including: recognising there was a need for systematic and ongoing education of staff and students to find connections to AI that linked to an enabling ethos; the realisation that some university SACO processes and practices needed to be revised within the enabling space; and the ongoing collegial work and discussions with UCO. This need to consistently educate staff is reinforced by literature that sees integrity issues as “a delicate dance”, negotiating a wide range of values, interests and obligations and where there is a need to provide tools and procedures to “enforce the stakeholders’ obligations to the institution” (Amigud and Pell, 2021, p. 940). In addition, education of staff is imperative given that “faculty members influence expectations and behaviors of students within their classes” (Hulsart and Mc Carthy as cited in Amigud and Pell 2021, p. 930).

In this paper, we reflect upon and evaluate the effectiveness of our approach across UCO and UoNC using the the five core elements of AI culture which are typically held to exemplify an ‘educative’ approach to AI: ‘support’, ‘access’, ‘approach’, ‘responsibility’ and ‘detail’ (Bretag and Mahmud 2016). Quantative data was used to inform and support the qualitative thematic analysis.

### Limitations

While we recognise a major limitation to this study is the absence of qualitative instruments such as surveys and interviews with student participants, anecdotal observation of student responses to their email invitations and increased attendance at the meetings, was one indication of determining if our educative approach had a positive effect on students’ engagement with the investigation process. This study provides only two Enabling program experiences in an Australian higher educational context. This limits the concrete conclusions that can be drawn between specific interventions over a number of years. It may be argued that such AI practices mentioned are widely acknowledged to be best practice in recent years. However, with limitations in the exact impacts, these case studies still highlight how this critical educative approach correlates with a cultural shift in AI.

### An educative culture of AI

Besides specific teaching and learning activities, research has shown that a whole systems approach is needed to foster a culture of AI amongst students, staff, and administrators (see Gallant 2008, 2011; Davis, Drinan, and Gallant 2009; Macdonald and Carroll 2006; Sutherland-Smith 2008; Bretag et al. 2014). This approach is described in Bretag et al.’s seminal report on exemplary practices in AI policies. They note that effective and educative AI policies need to focus on: “Access, Approach, Responsibility, Support and appropriate amount of Detail” (Bretag and Mahmud 2016, pp. 473–474). ‘Access’ refers to a “policy [which] is easy to locate, easy to read, well written, clear and concise. The policy uses comprehensible language, logical headings, provides links to relevant resources and the entire policy is downloadable as in an easy to print and read document” (p. 473). In ‘Approach’, exemplary AI policies view AI “as an educative process and appears in the introductory material to provide a context for the policy. There is a clear statement of purpose and values with a genuine and coherent institutional commitment to academic integrity through all aspects of the policy” (p. 473). In terms of ‘Responsibility’, “the policy has a clear outline of responsibilities for all relevant stakeholders, including university management, academic and professional staff, and students” (p. 473). ‘Support’ entails “systems are in place to enable implementation of the academic integrity policy including procedures, resources, modules, training, seminars, and professional development activities to facilitate staff and student awareness and understanding of policy” (p. 474). Finally, sufficient ‘Detail’ ensures “[p]rocesses are detailed with a clear list of objective outcomes, and the contextual factors relevant to AI breach decisions are outlined. The policy provides a detailed description of a range of AI breaches and explains those breaches using easy to understand classifications or levels of severity. Extensive but not excessive detail is provided in relation to reporting, recording, confidentiality and the appeals process” (p. 474).

Bretag and Mahmud (2016, pp. 467–477) highlighted the value of an AI culture or ethos within institutions. They identified six components contributing to AI culture: “academic integrity champions, academic integrity education for staff and students, robust decision-making systems, record keeping for evaluation, and regular review of policy and process”. This culture heralds a paradigm shift from misconduct to integrity and focuses on working with students as partners.

In the following case studies, we draw from the above-mentioned work of Bretag and Mahmud (2016). As AIOs and SACOs at UCO and UoNC, we have taken extensive steps to ensure that our AI policies and processes are transparent, accessible and comprehensible to encapsulate a holistic culture of AI. In this paper, due to the specific needs of the enabling cohort highlighted earlier, our focus is to specify how we foster and embed an educative culture of AI through supportive and inclusive pedagogies. Thus, our attention in this paper is on the elements of ‘support’, ‘approach’ and ‘responsibility’. In the following case studies, we demonstrated how our work as AI Champions reflects a change of culture in AI education.

## Discussion and reflection

This section outlines the various strategies implemented at both programs to enhance students’ learning of AI and describes how the investigation and management of cases have been modified. As mentioned previously, a limitation of the study is the absence of student surveys and interviews, and therefore we rely upon our own practitioner experiences and observations with the students as their AIOs and SACOs. We reflect upon these experiences and anecdotal accounts to evaluate the effectiveness of our approach.

### Educative and enabling approach at University of South Australia College and University of Newcastle, English Language and Foundation Studies Centre: expanding on the existing AI policy

At UCO and UoNC, we have adopted a whole systems approach to foster an educative culture of AI amongst students, staff, and administrators.

### Approach

At UCO and UoNC, we take an educative approach which aligns with our institutions’ policies for AI case management and procedures. Our approach has been recognised for its positive impact in supporting students’ understanding of AI practices. This was formally recognised with a commendation in TEQSA’s 2017–2018 AI report^1^ and an institutional and national teaching citation award (Universities Australia 2019). Our collaborations with other AI champions (called Academic Integrity Officers (AIOs) at The University of South Australia and Student Academic Conduct Officers (SACOs) at The University of Newcastle) show a commitment to the institutional values of AI. An educative approach involves fostering an inclusive learning environment for the diversity of the enabling student cohort. Students enrolled in our programs represent various cultural and language groups with noticeable variation in language and literacy proficiency. In addition, our students can often feel intimidated in their acculturation to a new academic environment. For example, UCO^2^ reports that 75% of College students represent the ‘first in family’ to attend university. Further it is common for students to exhibit low levels of self-confidence due to past negative educational experiences at school. At UoNC, 25% of students in enabling education self-reported as having a disability^3^ which, according to the literature (e.g., Grimes et al. 2017) can have a significant impact on students’ engagement with academic culture. Due to these factors, we recognise entering university and reengaging with study, sometimes after a long absence, can be quite daunting for enabling students. Therefore, emphasizing an educative approach to AI in institutional policy is integral.

Our educative approach is also grounded in research that suggests a shift towards explicitly unpacking the cultural expectations of the HE system including AI (see Grira and Jaeck 2019; Picard et al. 2018; Fishman 2015; Sutherland-Smith 2014). These considerations include fostering a culture of AI and a “reflective approach” which “calls for mindfulness, empathy, and skilful dialogue on the part of the instructor and appears to encourage critical self-reflection in the student” (Dalal 2015, p. 1). Cognisant of our students’ inexperience with university systems and discourse, we provide respectful guidance to assist their understanding of AI policy. Respectful guidance, for example, involves explaining AI nomenclature such as, ‘*academic integrity policy breach*’, ‘*plagiarism*’ and ‘*academic misconduct*’. Some of these terms connotate negative and serious implications and thus our alternative approach is preferred. In earlier AI meetings, several students reported difficulties in interpreting the language in official AI correspondence finding it intimidating and punitive with use of words such as ‘*being charged with plagiarism’*. Therefore, we adjusted the wording of some phrases ensuring consistency with the official policy but demonstrating our respectful guidance for students’ learning.

Our universities’ educational approach emphasizes an ‘ethos of care’, where support, as well as encouragement is part of enabling pedagogy. Because some students in Enabling programs have experienced education as a gatekeeper and they may lack the social and cultural literacy skills necessary to navigate their way through the complex university terrain, we work on fostering ‘ethos of care’ in order to enhance a student’s sense of belonging. We recognize that support mechanisms are necessary to build upon student understanding of AI practice and processes.

### Support

The two elements of approach and support are intertwined. In order to be reflexive to our students’ various learning needs, providing a range of supports is essential. Further, academic staff require support and examples of how to include AI content into their teaching. Following the advice of AI research (Bretag and Mahmud 2016; Morris 2015; Dalal 2015) we have created AI learning resources and suggested ways to interweave them into the program curricula. At UCO, we have developed and facilitated four workshops each semester to teach students academic literacies that are integral to enhancing their understanding of AI. In the workshops, students are taught conventions such as: summarising, paraphrasing and understanding referencing mechanics to avoid being implicated in breaches of AI policy. The central aspects of the workshops are reinforced in the core academic literacies course called, University Studies, ensuring that AI processes and academic conventions are thoroughly explained to all students. Embedding AI content in the curriculum of core units is particularly significant for students’ learnng of ethical academic behaviour. As Morris (2015, p. 1) argues, incorporating formative activities and scaffolded examples of AI literacies presents opportunities for students to receive feedback and guidance from teaching staff and peers. In our experience, student feedback has indicated the usefulness of embedding these supports within the curriculum:

Referencing was a big issue that I struggle with, however this course has helped me extremely … the best aspect of the course was providing understanding of what is expected in summaries, paraphrasing etc. also understanding how to better use words and sentences.^4^

At UoNC, there is also an emphasis on embedding the university AI processes into the enabling pedagogies. Students receive support and scaffolding to complete the University’s Academic Integrity Module and enabling SACOs work with others in the university in a consistent way. The focus within the Enabling program at The University of Newcastle is to ensure that staff embed AI requirements and support into each assignment. In 2018 and 2019, workshops were held where staff reviewed key summative assignments and existing resources to unpack the AI requirements and communicate expectations in simple language that the students could understand. This curricula is complemented with workshops and materials developed by Academic Advisors who support students outside of class individually and in groups and present short sessions in class on request of the lecturers.

We know that students entering Enabling programs may not have the requisite strategies in place leaving them to feel isolated and overwhelmed (Forsyth and Furlong 2003). Therefore, as AI champions (AIOs and SACOs), we are supportive of the identity shift students undergo in their induction to university life. Encouraging conversations and feedback between students and staff in relation to AI processes has resulted in the revision of policies to suit the diversity of our student cohort. Teaching faculty are encouraged to discuss their concerns with us so we can then advise ways to improve scaffolding and make amendments to assessments to minimalize AI issues. As AIOs and SACOs, we determine reasonable outcomes following investigation of misconduct, and these are always educative, allowing the students opportunities for further feedback and in some cases resubmission of their work. Due to these open conversations between staff, students and the AIOs/SACOs, changes have occurred to support teaching and the AI processes themselves. For example, at UoNC, the SACO keeps a list of common issues encountered by teaching staff. These accounts have provided relevant material for the development of short five-minute self-contained modules. Each module includes reflective questions focusing on different AI scenarios. These resources, available for the benefit of both staff and students, can be embedded into teaching materials or used for assessment preparation. The brevity of these resources also assists staff members who tend to avoid discussing AI issues as they believe it to be too time consuming (MacLeod and Eaton 2020).

At both institutions, the text-matching software, *Turnitin*, is used for detecting cases of academic misconduct and providing evidence for AI investigations. There is a responsibility in teaching students and staff to use and understand the generated reports to maximise successful learning outcomes. Both UCO and UoNC, provide workshops and online resources which are specifically designed to support students to interpret *Turnitin* reports and teacher feedback. These supports encourage students to view *Turnitin* as an educational tool, rather than software that flags students for punitive repercussions.

At UCO, communication with students has shown us they are often intimidated by AI processes. Hence, as part of this enabling educative approach, we show our students an educational video developed to dispel the stigma surrounding AI investigations. The video presents a role-play between two students as they discuss the most common concerns students have about AI breaches. We include this student-centred resource in our initial email contact to students suspected of AI breaches to alleviate feelings of anxiety. The video is effective at providing empathetic guidance and advice for students. This is evidenced by student comments: *The video made me feel at ease and comfortable … . It eased my mind about the meeting.*^5^ This resource has inspired the creation of other AI focused videos for the Oxford University Press Epigeum (a leading provider of online courses that support student learning and well-being) accessible for the international university community.^6^ Due to the successful impact the video has had on improving the transparency of AI investigations and processes, other academic divisions have shown interest in UCO’s supports and educative approach to AI. The video is an exemplar of student centred practice and has inspired conversation amongst the AI champions at this institution to generate a suite of innovative support resources to help students engage with AI processes rather than shy away from them. At UoNC, a video with the SACO explaining the process and calming student concerns has also been added to the AI breach notifications the students receive via email. In this way, complex issues around breaches of AI policies are addressed as democratically and supportively as possible.

At UCO, we have adapted a version of *The Academic Integrity Board Game* (White 2018) which we play in the University Studies (core course) tutorial classes. This gamified AI activity is tailored with hypothetical scenarios and questions related to AI experiences at university and beyond. The conversations that arise during gameplay explore students’ understandings and perceptions of what is considered ethical academic behaviour. Both students and tutors have found this game is another useful activity to create awareness and discussion about AI.

In line with our educative approaches, we believe that it is important to help the student get to the root cause of why an AI breach occurred initially. It may be due to a variety of reasons such as: troubles in understanding the requirements of the assessment task, difficulties experienced with academic language, challenges with balancing study, work and life commitments and mental health/anxiety issues. Overall acceptance of diversity is a central premise of our supportive enabling educative approach. Recognition of our diverse student cohort implies that we acknowledge and accept that there are many complex factors which can derail one’s study progress and compromise academic conduct. Because the causes of AI breaches are often multifaceted, students are provided with a range of supports to accommodate their learning needs. For example, a student may be referred to online resources or workshops to help them revise institutional AI policy guidelines, or if an EAL/D student inadvertently plagiarises or poorly paraphrases due to language proficiency limitations; they may be referred to a learning adviser for help with academic writing. If the cause of the breach is related to external, personal issues which may distract the student from their studies, a referral to a counsellor or program director might be necessary. These examples demonstrate that supports need to be tailored to students’ individual circumstances.

### Responsibility

Responsibility entails all relevant stakeholders (students, academic and professional staff) are aware of their role in upholding and fostering an educative culture of AI at “individual, organisation, education system and social levels” (Bretag et al. 2011, p. 4). Providing clear and transparent guidelines in institutional policy enables stakeholders to understand their responsibilities and remain accountable for embedding a culture of AI in their daily work. Because of the diverse learning needs of enabling cohorts, practitioners are mindful of their responsibilities to embed AI literacies and values within their pedagogy and curriculum.

UCO’s institutional Assessment Policy and Procedures Manual (APPM) indicates that all AI cases will be managed as an educative process for students. This includes: “formative opportunities for practice” as well as educating staff about “assessments that minimise misconduct” (APPM 2021, p. 44). At UCO, we adhere to these aspects, by ensuring curriculum is scaffolded and flexible to incorporate new resources and activities. Given our students represent a range of backgrounds and experiences, we work on enhancing democratic participation in AI processes where student feedback shapes our approach. This has informed the creation of resources that provide students with clarity about AI processes. Furthermore, at UCO, AI is promoted as the responsibility of both staff and students. Staff are encouraged to participate in professional development activities to broaden their understanding of AI processes and contribute to the overall values and culture of AI. Workshops with AI resource PowerPoint presentations and online modules are provided for local and regional staff so the process and approach is centralized and consistent across university campuses. Staff such as course coordinators, lecturers and especially sessional tutors, who may have the most contact with students, are critical in embedding this educative ethos surrounding AI.

Respectful guidance ties in with the element of ‘responsibility’. As a part of our enabling pedagogical approach, students are guided and encouraged to take charge of their AI learning. At UCO, we find that peer and collaborative learning is necessary for fostering a culture of AI. To encourage students to attend the AI investigation meetings and assure them that they are not alone, we restructured the process from individual to group interviews. This has proven to be a more successful strategy for engaging students in open and educative discussions about AI learning than what was achieved when interviewing students on their own. Group consultations for first-time AI breaches (of a similar nature e.g. poor paraphrasing or referencing issues) helps to alleviate student anxiety and demonstrates that they are not being targeted individually. In these meetings, students are shown how to check their *Turnitin* reports and access AI online resource modules to prevent them from breaching conduct again. Group investigations also encourage students to share what they have learnt via word of mouth enhancing the peer-learning process.

At UoNC specific AI Peer Assisted Study Support (PASS) sessions have assisted students in educating each other around aspects of AI. Students become increasingly responsible for themselves and can reflect on their errors and their peers’ learning. As SACOs, we advise teaching staff and Associate Deans of Learning and Teaching on how and where to improve and update their assessments to further avoid AI. This improved feedback loop between us and staff ensures reporting of possible breaches and acknowledges the responsibility between all stakeholders.

### Outcomes

Enabling students’ inexperience with academic literacies and conventions can lead to inadvertent indiscretions such as plagiarism or a lack of referencing acknowledgement, which is usually detected in the submission of their first assessments. This can account for the high number of AI breaches identified in the early stages of enabling students’ study programs. While the high number of cases may appear alarming, we have found that identifying breaches early and providing students with academic counselling is a necessary and educative step in preventing students from breaching again. UCO has demonstrated that early interventions and embedding of supportive learning resources in the Colleges’ curricula has enhanced students’ understanding of AI and has influenced a noticeable reduction in repeat breaches. Since 2016, the AIOs approach to fostering an educative culture of AI has affected a 20% reduction in misconduct cases. From 2016 to 2020, 268 students who were counselled for AI have transitioned into undergraduate degrees. Of those, only five students (1.8%) have been investigated for misconduct a second time. This data shows a correlation between UCO’s approach and a sustained impact on students’ understanding of AI.

Upon reflection of our approach, we argue an ethos of AI needs to be constantly reinforced through pedagogy and embedded in the curriculum by providing activities, scaffolding and examples of how academic literacies are employed in different text-types (Picard et al. 2018; McGowan, 2005). This reflects Bretag and Mahmud's (2016, p. 473) AI model’s core element of ‘approach’. Our study has shown that processes which are centralized and consistent across university campuses are important for building a responsible culture of AI. Creating curriculum resources for staff has ensured that teaching staff do not feel overwhelmed with referring cases and updating AI learning materials.

At UoNC there were 32 AI cases reported in 2017. This steadily increased to 93 cases in 2018 and 155 cases in 2019. Other variables, such as an increase in the student cohort and more online and take-home exams may account for some of this increase. However, through analysis of feedback by staff members, it has been found that the increase in cases has been largely due to continual education by the SACOs gradually changing the attitudes of staff members towards reporting cases and an increased ‘buy-in’ to the educative process. There were a great many misconceptions amongst teaching staff around the management and reporting of suspected AI cases. Misunderstandings included that reporting was a choice. Further, some teaching staff felt there was an ethos ‘to protect’ students from the potential consequences of an investigation and were at liberty to determine outcomes for themselves. This mirrors other current research where academic staff are seen to be hesitant or unwilling to report cases or engage in discussion related to AI (MacLeod and Eaton 2020). They may be also be “tempted to exercise their own version of integrity grounded in a personal sense of fairness, justice and duty” (Amigud and Pell 2021, p. 929).

As teaching staff began to realise that the AI process was, in fact, predominantly an educative one and that there were legalities of taking academic matters into their own hands, the number of cases referred to the SACO grew exponentially. The majority of cases were, and still are, linked to plagiarism (frequently through lack of understanding of ‘the rules of the university game’, difficulty with paraphrasing and/or poor time management and ‘panic’) with some self-plagiarism, collusion and direct copying of examples and templates supplied.

### Challenges

A challenge encountered at both institutions has been encouraging academic staff to re-think how they design their assessment tasks. Investigating large numbers of students for misconduct relating to the same assessment can indicate the design of the task or the instructions provided are not clear. For example, courses which have traditionally assessed students’ performance through invigilated examinations saw a rise in breaches during the COVID pandemic when the assessment mode was switched to online. There has been a conflict in understandings with students assuming online assessment meant it was acceptable to collaborate with peers and look-up answers online, while academic staff expected students to continue working independently. In response, as AIOs and SACOs, we have had ongoing discussions with staff suggesting ways to change the instructions so that the expectations around assessment security can be more clearly understood. Further, we have encouraged staff to re-consider their design of online assignments and exams to reduce the potential of academic misconduct. This, as well as staff spending more time educating students around AI issues with our encouragement and assistance, may have had an impact on the number of cases reported in 2021 thus far. At UoNC, there have been 35 cases reported from the beginning of 2021 until the end of April which is significantly less than reported for this timeframe in 2018 to 2020. The conclusions drawn from the outcomes were reached through informal conversations and written feedback of staff members, stronger collaboration between staff and students and through increased discussions with students via Zoom and other platforms.

Zoom has helped the AIOs and SACOs maintain their educative approach to investigation, however one complication has been encouraging students to turn on their cameras and to use a device with a large monitor to screen share. Throughout the interview with the student, we draw upon examples of the students’ work and suggest ways for how they could better incorporate referencing, paraprahsing and meet the academic requirements of the task. It is difficult to do this if the student is using a mobile phone device with a small screen which limits what they can see. It can be even more distracting if the student is joining the meeting from a remote location or on public transport when background noise can carry over the conversation. Further, when students are reluctant to turn on their cameras, it can be hard to determine the intention of the misconduct as body language can be a useful indicator. For these reasons it is always best to conduct the investigations in a face-to-face meeting when possible.

While COVID-19 brought with it many challenges, including an increase in academic misconduct in assignments using digital technology and the need for AI to be reviewed for “a digital world” (Reedy, Pfizner, Rook and Ellis 2021, p. 23) it does appear to have impacted positively on the number of students who had extensive conversations with us regarding AI issues. There seemed to be more of a readiness to discuss with us online than face to face consultations and meetings.

### Further research

As mentioned earlier in the paper, students in Enabling programs can face several challenges in their induction to academic culture including inexperience with academic literacies and conventions, time management and other external pressures*.* Futher, the attrition rate in Enabling programs is concerning with approximately 50% of students administratively withdrawing and/or dropping out of the programs (Hodges et al. 2013). Some of the students who leave the program have also been called up for AI investigations. To build upon anecdotal evidence, further investigation into understanding why students in Enabling programs are implicated in academic misconduct is needed. Ultimately, this may also provide some context for why students inevitably leave the program. If these issues are flagged at the investigation process, the students could receive academic counselling and be referred to supports they require.

Recently, the Australian government introduced the Job-ready Graduates package of HE policy reforms (Department of Education, Skills and Employment 2020) with the aim to steer student enrolments towards courses which will generate better employment outcomes. The package will increase the number of Commonwealth (Government) Supported Places in disciplines deemed a national priority. The policy also proposes plans to remove financial support for students who fail half of the courses studied in their first year. Research (e.g. Grant-Smith, Irmer and Mayes 2020) has shown that students from equity groups, which is generally what the enabling student cohort is comprised of, have not performed as well as their non-equity group peers in their undergraduate and postgraduate study. Under these new reforms, it is imperative for HE institutions to demonstrate how they will support these groups of students (Grant-Smith, Laundon and Feldman 2020) or inequity of access is likely to increase. Therefore, it is now even more important to support students in Enabling pathway programs and equity group students in all programs so that they feel more confident with their understanding of AI literacies early in their academic careers. Further research on the potential of a combination of enabling and educative approaches would therefore be invaluable to ensure the retention and success of these students.

## Conclusions

While methods of detecting AI breaches may increase and become more sophisticated, it is important that a proactive and educative approach to AI is taken to change culture overall. The widening participation agenda has successfully increased the number of equity group students in HE. This evolving student cohort represents diverse educational backgrounds and experiences. Therefore, a move from a punitive to an educative approach to AI is necessary to build upon a new cultural capital for university study. For this reason, the educative AI approach we have taken in these Enabling programs is a successful model for all university degree programs regardless of year level. Measures to improve AI must involve all stakeholders including students, staff, and the wider university to proclaim a true culture shift.

Given that causes of AI breaches are often multifaceted, we recommend that student supports are tailored to the individual. From our perspective, the process of supporting individual needs and circumstances is ongoing given the student cohort varies from year to year. As can be seen by the innovations mentioned in this paper, change does take time and effort e.g. it took three to six months to be able to record and see small changes occurring in the process at UoNC. When academic staff are reluctant to refer cases on for investigation because of pressure to generate successful academic results, students go unnoticed without receiving the necessary guidance and support they need before transitioning into further studies. Hence, this is why it is important for all staff to respect and advocate the AI process. A challenge in establishing a systemic approach is the turnover of staff, particularly sessional teachers, who require ongoing education and induction into AI management and processes.

As educators we must ensure we provide a curriculum that is scaffolded and flexible to incorporate new resources and activities. Further, assessments need to be reviewed and redesigned to minimise opportunities for academic misconduct. It is here that AI becomes a holistic endeavour where embedded approaches that are clear and transparent are critical to enact lasting and positive change in the HE sector and beyond.

## Data Availability

Data regarding the number of academic integrity cases investigated was sourced from academic integrity records at each university. Due to the sensitive nature of this data, it cannot be shared as per institutional policy.

## Abbreviations

AI: Academic Integrity
AIO: Academic Integrity Officer
APPM: Assessment Policy and Procedures Manual
EAL/D: English as an Additional Language/Dialect
EFTSL: Equivalent Full-Time Student Load
PASS: Peer Assisted Study Support
HE: Higher Education
SACO: Student Academic Conduct Officers
SES: Socio Economic Status
TEQSA: Tertiary Education Quality and Standards Agency
UCO: University of South Australia College
UoNC: University of Newcastle, English Language and Foundation Studies Centre

## Footnotes

1 Tertiary Education Quality and Standards Agency 2018, *TEQSA Annual Report 2017–18,* Australian Government.

2 The University of South Australia, 2018, *Business Intelligence*, Australia.

3 The University of Newcastle, 2019, *Student Data*, Australia.

4 The University of South Australia, 2016–2018, *MyCourseExperience,* The University of South Australia, Australia.

5 The University of South Australia, 2018, Student Feedback, Australia.

6 Oxford university Press Epigeum, epigeum.com
